# Control of cell proliferation by a porous chitosan scaffold with multiple releasing capabilities

**DOI:** 10.1080/14686996.2017.1406287

**Published:** 2017-12-01

**Authors:** Shu-Jyun Cai, Ching-Wen Li, Daphne Weihs, Gou-Jen Wang

**Affiliations:** ^a^ Graduate Institute of Biomedical Engineering, National Chung-Hsing University, Taichung, Taiwan; ^b^ Department of Mechanical Engineering, National Chung-Hsing University, Taichung, Taiwan; ^c^ Faculty of Biomedical Engineering, Technion-Israel Institute of Technology, Haifa, Israel; ^d^ Tissue Engineering and Regenerative Medicine, National Chung-Hsing University, Taichung, Taiwan

**Keywords:** Porous chitosan scaffold, fibroblast growth factor, transforming growth factor-beta 1, wound dressing, controlled drug release, 20 Organic and soft materials (colloids, liquid crystals, gel, polymers), 102 Porous / Nanoporous / Nanostructured materials, 211 Scaffold / Tissue engineering / Drug delivery

## Abstract

The aim of this study was to develop a porous chitosan scaffold with long-acting drug release as an artificial dressing to promote skin wound healing. The dressing was fabricated by pre-freezing at different temperatures (−20 and −80 °C) for different periods of time, followed by freeze-drying to form porous chitosan scaffolds with different pore sizes. The chitosan scaffolds were then used to investigate the effect of the controlled release of fibroblast growth factor-basic (bFGF) and transforming growth factor-β1 (TGFβ1) on mouse fibroblast cells (L929) and bovine carotid endothelial cells (BEC). The biocompatibility of the prepared chitosan scaffold was confirmed with WST-1 proliferation and viability assay, which demonstrated that the material is suitable for cell growth. The results of this study show that the pore sizes of the porous scaffolds prepared by freeze-drying can change depending on the pre-freezing temperature and time via the formation of ice crystals. In this study, the scaffolds with the largest pore size were found to be 153 ± 32 μm and scaffolds with the smallest pores to be 34 ± 9 μm. Through cell culture analysis, it was found that the concentration that increased proliferation of L929 cells for bFGF was 0.005 to 0.1 ng/mL, and the concentration for TGFβ1 was 0.005 to 1 ng/mL. The cell culture of the chitosan scaffold and growth factors shows that 3.75 ng of bFGF in scaffolds with pore sizes of 153 ± 32 μm can promote L929 cell proliferation, while 400 pg of TGFβ1 in scaffolds with pore size of 34 ± 9 μm can enhance the proliferation of L929 cells, but also inhibit BEC proliferation. It is proposed that the prepared chitosan scaffolds can form a multi-drug (bFGF and TGFβ1) release dressing that has the ability to control wound healing via regulating the proliferation of different cell types.

## Graphical abstract

**Figure:**
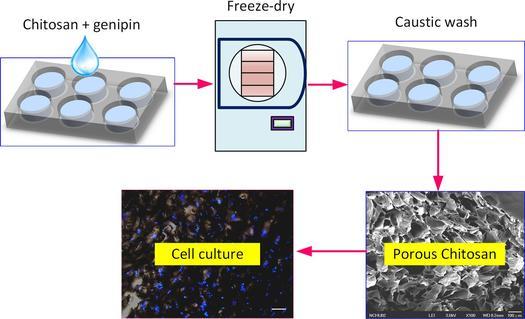

## 1. Introduction

The skin is the body’s largest organ, and its main functions are to regulate body temperature, prevent water loss, and inhibit harmful viral and bacterial infections from entering the body. However, damage to the skin from burns or cuts can cause significant loss of function; hence, it is desirable to treat skin damage as soon as possible. In the treatment of large areas of skin damage there are two methods, autologous and allogeneic transplantation, respectively, using tissue from the patient or from a different source. Autologous transplantations are preferable, yet both have shortcomings. Allogeneic transplantation has many issues, such as tissue rejection by the immune system, which complicates tissue repair and introduces difficulties and restrictions to this treatment method [1]. In autologous transplantation, the immunity problem is irrelevant, yet limited availability of replacement tissue reduces its utility [2]. Hence, the development of wound dressings in combination with tissue engineering has increased in recent years in an attempt to speed up the process of repairing and replacing damaged tissues.

Increasing interest in tissue engineering has also seen greater attention received for wound healing and dressing applications. Compared to traditional autologous or allogeneic transplantation therapies, the preparation of artificial wound dressings can provide large area coverage to damaged skin and also reduce the demand for donor tissue [1]. Wound dressings can also be loaded with active antibacterial, and hemostatic properties, as well as growth factors that promote the regeneration of skin [3]. Similarly, application of different stimuli, such as externally applied low-level stretching, can be used to accelerate closure of small, remaining gaps [4]. However, current artificial wound dressings that promote skin regeneration have been limited to the use of a single growth factor and the incorporation of multiple growth factors and other types of stimuli are rare; hence, the aim of this study is to develop a wound dressing capable of simultaneous release of multiple drugs [5].

Wound healing is a complex physiological process that involves the interaction of different tissues and types of cells, where the regeneration and repair time especially in skin are dependent on the area and depth of damage. The healing process of a skin wound can be separated into three major stages: inflammation, proliferation, and remodeling [6]. When skin trauma occurs, the first response by the body is a coagulation of the blood at the trauma site, followed by inflammation of the surrounding tissue. Neutrophils and adipose cells migrate to the damage site to remove infection and debris, with subsequent fibroblast and endothelial cell proliferation at the site to cover and heal the wound [7–11]. The inflammation stage of wound healing is to prevent bacterial infection and clear cell debris, while the proliferation and remodeling phases are relevant to repair and regeneration of the trauma site. Hence, it is has been shown that the secretion of extracellular matrix by cells involved in skin tissue repair is important for effective wound healing [7].

Growth factors play an important role in wound healing, which will affect the rate of healing and formation of scar tissue. For instance, fibroblast growth factor (bFGF) can stimulate fibroblastic proliferation, while transforming growth factor β1 (TGFβ1) can promote collagen formation [6,12]. It has been reported in the literature that the exogenous half-life of these growth factors is quite short and that they are easily degraded by enzymes *in vivo*; hence, one of the issues at hand is to effectively control delivery of growth factors to the target site through carriers [13].

Chitosan is a biocompatible material that has the ability to stimulate macrophages, promote cell proliferation, encourage coagulation, and block nerves to reduce pain at the trauma site [14]. Chitosan is a natural polymer with good biocompatibility, biodegradability, and antibacterial properties as well as the ability to promote blood coagulation, hence is often used as wound dressings [15]. There have been reports where chitosan has been used as wound dressings to promote fibroblast proliferation and can help organize collagen to increase the healing rate of the wound [14]. Porous chitosan scaffolds have been prepared through freeze-drying, solvent casting/salt leaching, phase separation, electrospinning and 3D-printing methods [16–20]. Porous scaffolds have been widely used in tissue engineering, as the porous structure is able to allow the migration and diffusion of cells and nutrients. In addition, cells seeded in porous scaffolds are able to migrate from the surface and enable the uniform secretion of extracellular matrix throughout the scaffold to effectively repair and regenerate the wound [21].

In this study, the authors develop and evaluate the release of incorporated growth factors, bFGF and TGFβ, in different porous chitosan scaffolds, fabricated via lyophilization. The chitosan scaffold and incorporated growth factors were then used to examine the effects of drug release on cell proliferation of fibroblast and endothelial cell lines. The aim of the current study is to aid in the clinical development of porous chitosan scaffolds in wound healing.

## 2. Materials and methods

### 2.1. Fabrication of porous chitosan scaffolds

A 0.6% (w/v) chitosan solution was prepared by adding powdered chitosan (Sigma-Aldrich, St. Louis, MO, USA) to 1 M of acetic acid. The chitosan solution was then placed into the wells of a 24-well culture plate (Corning®) with the addition of a cross-linking agent, genipin (Wako Chemicals), at a 0.25% (w/v) concentration, and left to incubate at room temperature for 24 h. Various cross-linked chitosan samples were then placed into respective −20 and −80 °C freezers for 1, 6, 12, 24, and 30 h. The chitosan samples were then removed from the freezers and lyophilized for 24 h at a temperature of −52 °C and pressure of 28 Pa. The freeze-dried scaffolds were then washed in 1 M of sodium hydroxide solution for 30 min, to stabilize the pH, and then washed with deionized water for another 30 min. Finally, the scaffolds were allowed to dry at 40 °C and stored for future use up to 2 weeks from preparation.

### 2.2. Material characterization

The surface morphology of the scaffold was observed with field emission scanning electron microscopy (FESEM, JEOL JSM-7401F), and the pore sizes were measured from the obtained SEM images by randomly selecting 30 holes in a field of view. The pore sizes were measured with ImageJ software.

### 2.3. Controlled drug release of porous chitosan scaffolds

In this study, the amount of growth factors used were 3.75, 7.5, 15, and 30 ng for bFGF and 50, 100, 200, and 400 pg for TGFβ1 for drug release experimentation. Firstly, the porous chitosan scaffolds were placed in 24-well plates and coated with 5% (w/v) gelatin solution and left to dry at room temperature. The growth factors, bFGF and TGFβ1, were then injected directly into the porous chitosan scaffold with a micropipette.

To evaluate the drug-releasing capability, 1 mL of culture medium (LDMEM) was added to each well containing the scaffold and placed into a 37 °C humidified CO_2_ incubator. The medium was collected at time intervals of 1, 4, 8, 12, and 24 h as well as at 3, 7, 14, and 21 days to determine the amount of growth factors that diffused into the surrounding medium. When the medium was collected at each time interval, it was replaced with fresh medium.

The amount of growth factor present in the medium was determined with human FGF-basic ELISA MAX™ Deluxe Kit (Biolegend®), and human/mouse TGFβ1 uncoated ELISA Kit (Invitrogen) according to the protocols provided.

### 2.4. Cell tolerance to growth factors

In this study, mouse fibroblast L929 cell line and bovine carotid endothelial cell line (BEC) were used in cell experimentation. Both cell lines were cultured in LDMEM (Gibco™) supplemented with 10% fetal bovine serum (Gibco™) and 1% antibiotic/antimycotic (Gibco™) in a 37 °C humidified CO_2_ incubator with medium refreshment every second day and passaging of cells with TrypLE™ Express.

The growth factor concentration was diluted from a stock solution of 50 μL/mL of bFGF and 1 μL/mL of TGFβ1. Diluted concentrations used for experimentation were 0.005, 0.001, 0.05, 0.1, 0.5, 1, 5, and 10 ng/mL, the same series of dilutions for both growth factors.

For cell tolerance testing, 90% confluent cells were trypsinized and seeded at a concentration of 10^3^ cells/well and cultured in medium supplemented with growth factors according to the above-mentioned concentrations. The supplemented medium was refreshed every second day, and cell proliferation and viability were evaluated on the seventh day.

### 2.5. Cell seeding on porous chitosan scaffold with growth factors

The porous chitosan scaffolds were first washed with 75% ethanol and then in phosphate-buffered saline (PBS) prior to placing in 24-well culture plates. The scaffolds were further sterilized with overnight UV irradiation. The chitosan scaffolds were coated with 5% (w/v) gelatin solution and injected with growth factors prior to cell seeding. Finally, L929 and BEC cells were trypsinized and seeded at a concentration of 5×10^3^ cells per scaffold. The cells were then incubated in a 37 °C humidified CO_2_ incubator over seven days prior to analysis.

### 2.6. Cell proliferation and viability

Cell viability was measured with cell proliferation and viability WST-1 reagent kit (BioVision). After seven days, the culture medium was first removed from the wells of the culture plate and replaced with 1:10 WST-1 reagent to culture medium solution and incubated in a 37 °C humidified CO_2_ incubator for 2 h. About 100 μL of the working solution was removed per well and placed into a 96-well ELISA plate and measured with an ELISA reader with a measurement wavelength of 405 nm and a reference wavelength of 595 nm. The absorbance values were then used to calculate the proliferation and viability of the cells.

### 2.7. Fluorescent staining

Cell distribution on the scaffold was observed using fluorescent staining with 4′,6-diamidino-2-phenylindole (DAPI). After seven days, the cells were fixed with 4% paraformaldehyde for 30 min, and then 0.1% of triton X-100 was added for 10 min for perforation. The non-specific blocking used 5% bovine serum albumin (BSA) for 30 min. Finally, 10000× dilution of DAPI was used to stain cell nucleus for 10 min. At each step, the sample was cleaned 3 times with phosphate-buffered saline. All procedures were performed at room temperature.

### 2.8 Statistical analysis

Statistical comparisons of the data were analyzed with one-way analysis of variance (ANOVA) combined with Tukey’s *post hoc* test with OriginPro 9 (OriginLab®).

## 3. Results and discussion

### 3.1. Fabrication of porous chitosan scaffolds

**Figure 1.**
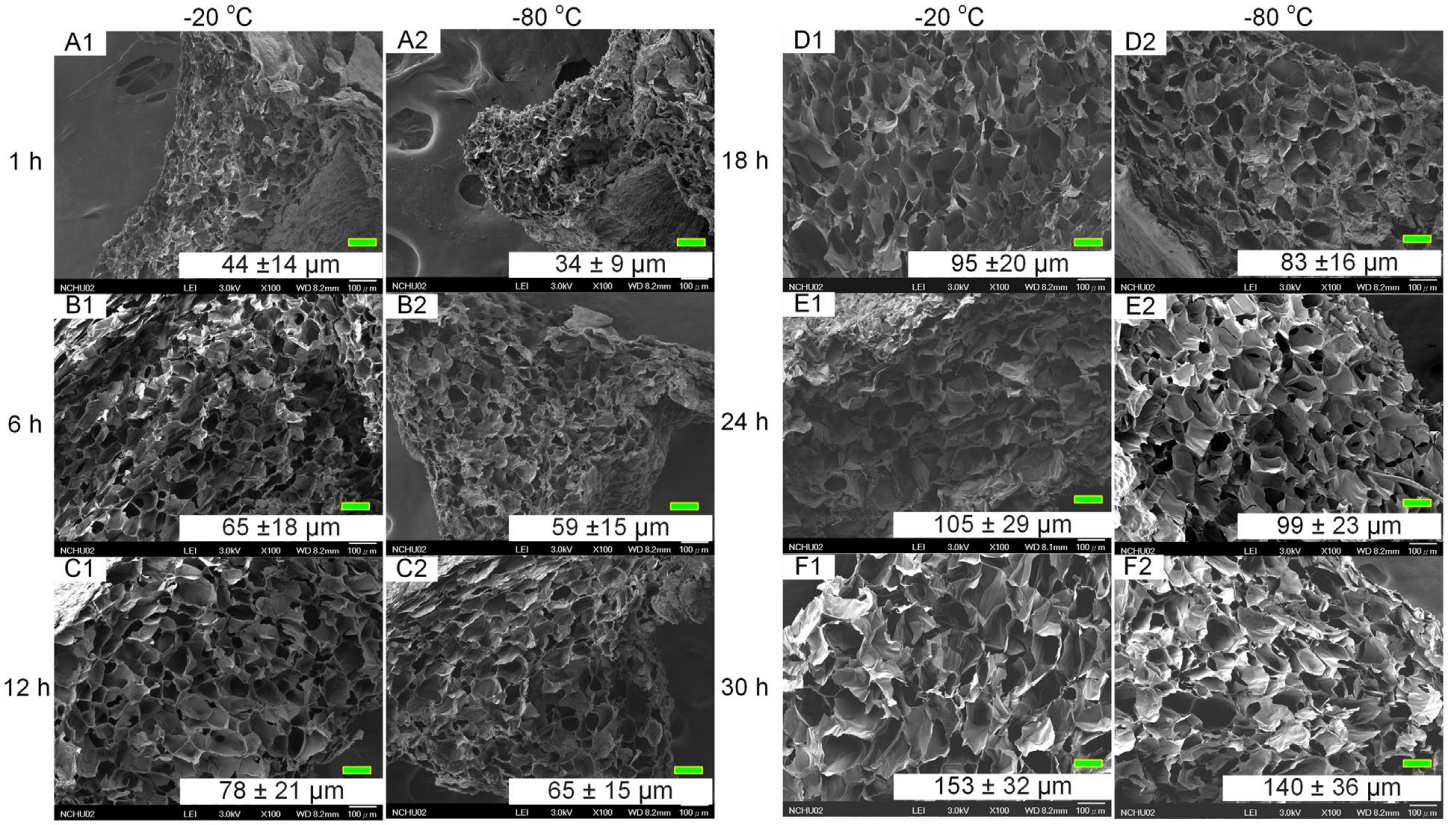
SEM images of porous chitosan scaffolds prepared at different freezing temperatures and times. Scale bar = 100 μm.

The porous chitosan scaffolds were prepared by using a freeze-drying method, where the solvent is crystallized via freezing and sublimated to form porous wound dressings. We used different pre-freezing temperatures and durations to investigate the effects of these parameters on the formation of the scaffold, specifically the pore structure and size. A concentration of 0.6% (w/v) chitosan solution was used in combination with pre-freezing temperatures of −20 and −80 °C and freezing times of 1, 6, 12, 18, 24, and 30 h; these were chosen to examine the effects of temperature and time on morphological changes to the porous structure (Figure 1). It was found that, regardless of the freezing temperature, the chitosan scaffold pre-frozen for 1 h showed similar pore structures with small circular and dense pores (Figure 1(a) and (g)). The pores increase with freezing time and appear to stack similar to mica sheets as shown in Figure 1(b), (h), (d), and (j) for freezing times of 6–18 h. As the freezing time reaches 30 h, the pore sizes are the largest, in comparison with other freezing times used in this study. In addition, the pore sizes are observed to be non-uniform and less dense. The results show that the pore sizes increase with freezing time and is consistent with the grain growth theory as well as previous reports that a longer reaction time will allow for greater sized grains to form [19,20].

**Figure 2.**
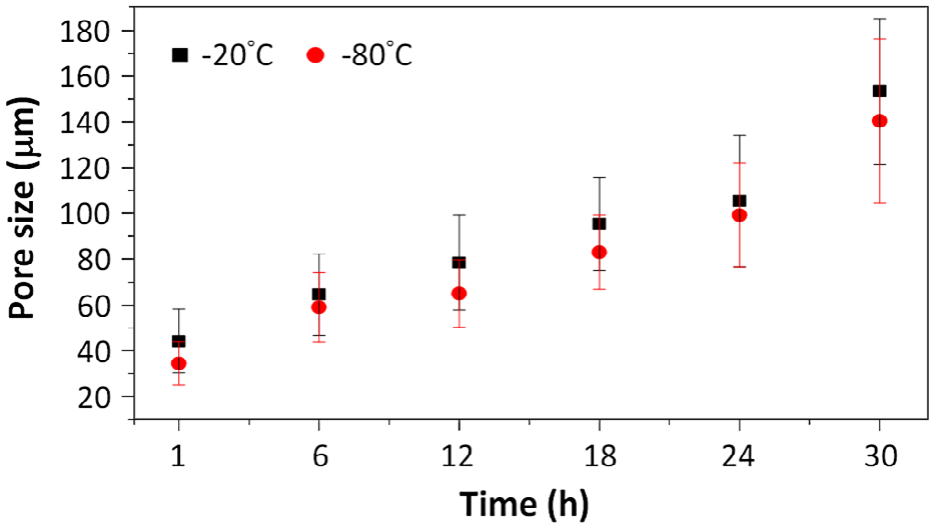
Pore sizes of the prepared porous chitosan scaffolds at different temperatures and times.

The pore sizes of the chitosan scaffolds formed at −20 and −80 °C with different times are summarized in Figure 2. The results show that the size of the pores is affected by both the temperature and time of pre-freezing. The pore size for −20 °C scaffolds is, on average, 1.06–1.28 times greater than pore size of scaffolds of −80 °C at the same pre-freezing time; as the freezing temperature is higher (−20 °C), the rate of solvent crystallization is slower resulting in larger crystals and, hence, larger pores [22,23]. However, the freezing time has a major effect on the final pore size, which is attributed to the growth of the crystals over time. The results show that the growth rate of the pore size is 3.2 ± 0.9 and 3.0 ± 0.8 μm/h for scaffolds prepared at −20 and −80 °C, respectively. It can be seen from the results that the pore size can be directly correlated to the freezing time and can be used to control the pore size of the scaffolds (Figure 2).

The Ostwald ripening theory [24] can be adopted to explain the pore formation process. In pure water, molecules do not freeze spontaneously at 0 °C, but form clusters that have the same water molecular arrangement as ice crystals and remain in liquid stage (supercooling). When some impurities (such as chitosan in this study) are present in pure water, they attach water molecules onto the surface to form ice nuclei and trigger ice nucleation. The temperature of ice nuclei formation usually varies between −2 and −15 °C, followed by growing crystal to become an ice crystal. There are three mechanisms to control crystal growth. The first mechanism involved in crystal growth is the growth from the perfect crystal side. The growth rate at the surface of which is controlled by surface nucleation rate. Screw dislocation is the second mechanism of crystal growth, and the ice crystal growth rate is related to the degree of interface supercooling. The third mechanism of ice crystal growth is called continuous growth, since the crystal is formed with large driving energy. In this mechanism, the nucleation obstacle does not exist, and growth rate is affected by freezing temperature. But, the crystal growth rate is proportional to the degree of interface supercooling. The maximum ice crystal generation temperature region is from 0 to −7 °C. When the time to pass through this temperature range is short, a fine ice crystal is formed. On the contrary, when the time is long, a large and rough ice crystal is formed [25].

In this study, the pore size was controlled by ice crystal growth, and we found that the pore size formed at −80 °C was smaller than those formed at −20 °C. The pore size increases with the freezing time. It was attributed to the time to pass through the ice crystal generation temperature range is short at −80 °C; hence, a small crystal was formed. But with prolonging freezing time, ice crystals tend to naturally arrange into large clusters via surface adhesion, sintering, and interlocking.

In this study, chitosan scaffolds with pore sizes ranging from 34.45 to 153.25 μm were fabricated, as it has been reported that pore sizes of less than 160 μm are suitable for the growth of human dermal fibroblasts. Larger pores result in increased penetration of the cells into the scaffold, while smaller pores promote cell migration [26,27]. Therefore, scaffolds (Pore I) with the largest pore size (153 ± 32 μm), produced at -20 °C and 30 h of pre-freezing, and scaffolds (Pore II) with the smallest pore size (34 ± 9 μm), fabricated at −80 °C and 1 h of pre-freezing, were used in evaluating the controlled release of bFGF and TGFβ1.

### 3.2. Analysis of L929 and BEC tolerance to bFGF

**Figure 3.**
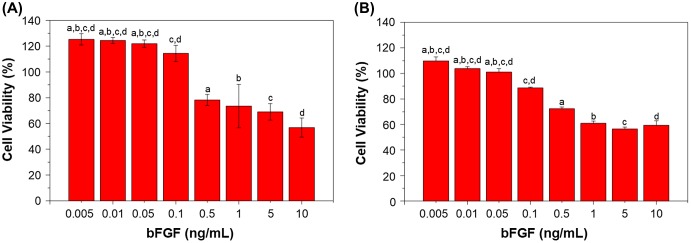
Viability of cells under different bFGF concentrations after seven days of culture; (a) L929 mouse fibroblasts and (b) Bovine endothelial cells. Viability in both cell types is shown relative to that of cells cultured on TCPS. Different letters indicate a significant difference when compared to the concentration labeled by that letter (*p* < 0.05).

The cell proliferation and viability of mouse fibroblasts L929 and BEC cultured in different bFGF concentrations on TCPS after seven days are shown in Figure 3, with percentages displayed relative to control cell culture on tissue culture polystyrene (TCPS). The results show that bFGF concentrations of 0.005 to 0.1 ng/mL promote proliferation of L929 cells, while concentrations between 0.005 and 0.05 ng/mL are able to promote BEC proliferation. When the concentrations exceed those mentioned above, an inhibitory response is observed, with lower cell viability [28,29].

### 3.3. Analysis of L929 and BEC tolerance to TGFβ1

**Figure 4.**
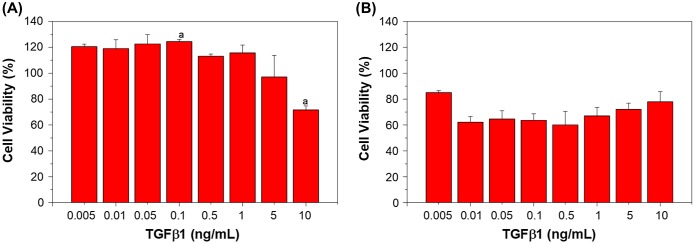
Viability of (a) L929 and (b) BEC cultured under different TGFβ1 concentrations for seven days. Results are shown in percent viability relative to TCPS. Letter *a* in (a) indicates a significant difference (*p* < 0.05).

In this study, the tolerance of TGFβ1 was also evaluated, as it has been reported that this growth factor is able to promote collagen production and contributes to wound healing and remodeling [30]. The cell viability of L929 and BEC cultured in different concentrations of TGFβ1 is shown in Figure 4, relative to TCPS control culture; it can be observed that TGFβ1 can enhance the proliferation rate of fibroblasts, and it was found that the tolerance range of L929 for TGFβ1 is greater than that of bFGF, with a range of 0.005 to 1 ng/mL compared to 0.005 to 0.1 ng/mL, respectively. In contrast, it was observed that endothelial cells are adversely affected by the inclusion of TGFβ1 into the culture medium, with even a small amount causing growth inhibition (Figure 4(b)). It has been reported that a combination of TGFβ1 with vascular endothelial growth factor (VEGF) can promote angiogenesis. In certain cases, increased angiogenesis rates cause the blockage of the lumen; hence, TGFβ1 is required to inhibit endothelial cell expansion to form the lumen [31]. Hence, in this study, a combination of TGFβ1 and bFGF was used to stimulate cell cultures to evaluate the effect of the two acting growth factors.

### 3.4. Controlled release of fibroblast growth factor-basic (bFGF)

**Figure 5.**
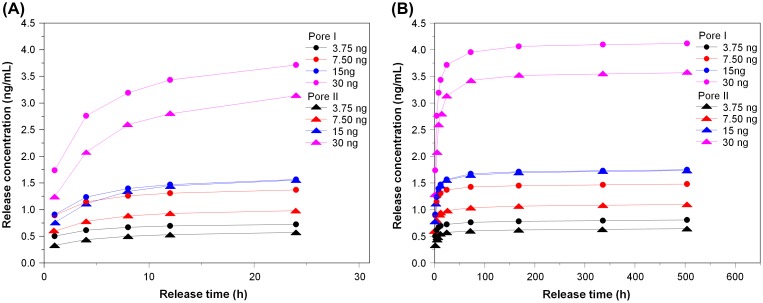
The cumulative release of various bFGF contents from porous chitosan scaffolds within (a) 24 h and (b) up to 21 days (1, 4, 8, 12, 24 h, and 3, 7, 14, 21 days).

**Figure 6.**
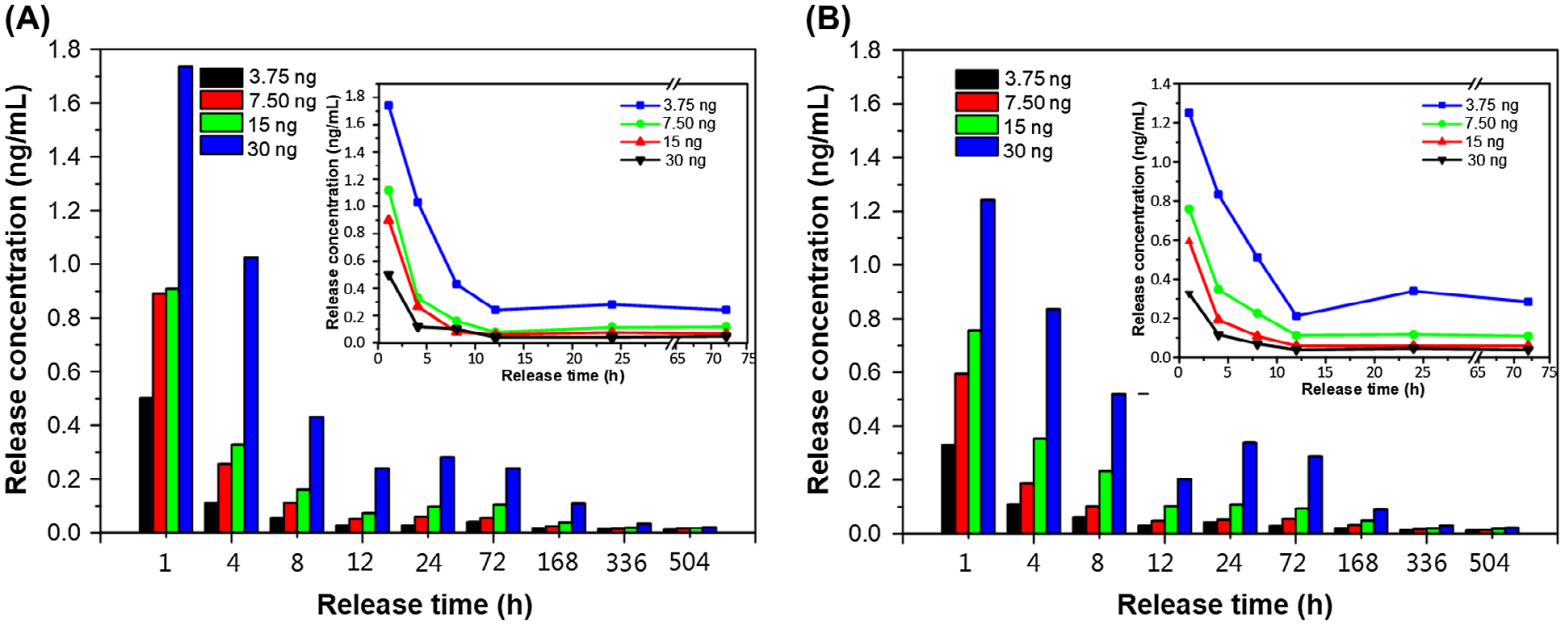
Single-point release of different, initial loading amounts of bFGF at different times following immersion in a solvent for (a) Pore I and (b) Pore II chitosan scaffolds. Inset: reduction trends for different loadings.

The porous chitosan scaffolds used for the drug release study were Pore I and Pore II, which were injected with 3.75, 7.50, 15, or 30 ng of bFGF to determine the release profiles over 24 h and also over the course of 21 days. The purpose of this test was to select the most appropriate bFGF injection concentration for cell culture. The cumulative release of bFGF is shown in Figure 5, and the time-dependent concentration in the surrounding media (single-point release) is shown in Figure 6. The reduction trends for different loadings are shown in the insets. It was found that a large amount of the growth factor was released within the first 24 h, regardless of scaffold, with an upper limit being reached on the third day (72 h). It was also found that the total amount and release rate of Pore I scaffolds were greater than Pore II, regardless of the initial bFGF content.

Table 1 details the release characteristics of Pore I and Pore II scaffolds at different initial bFGF content. From the initial bFGF content of 3.75 ng, it was found that the release rate within the first 24 h was 15.06 and 11.90 pg/h for Pore I and Pore II, respectively, which equates to 4.02% and 3.17% of the total bFGF content. This observed behavior may have been due to bFGF contained on or near the surface of the scaffold to be rapidly released, resulting in the observed release rates. It can be seen from Figure 5 that from the third day onwards the release rate becomes steady due to diffusion from within the scaffold, with an average release rate of 2.06 and 1.78 pg/day for Pore I and Pore II, respectively. The total amount of bFGF released over 21 days (504 h) was measured, and it was observed that 402.59 pg and 321.11 pg of bFGF were released for Pore I and Pore II, respectively, which equates to 10.74 and 8.56% of the total content. The results indicate that the scaffold with the larger pore size will have greater diffusion rates, resulting in greater bFGF content released. In addition, when the initial bFGF content was increased up to 30 ng, the total amount released also increased, as a greater concentration difference will result in greater diffusion rates; the effect of the concentration dominates the effect of the pore sizes in regard to diffusion rates.

**Table 1.** Release characteristics of various bFGF contents from porous chitosan scaffolds.

bFGF content (ng)	Average release within 24 h (pg/h)	Average release from day 1 to day 21 (pg/day)	Cumulative total over 21 days (pg)
Pore I			
3.75	15.06 ± 0.02	2.06 ± 0.10	402.59 ± 0.04
7.5	28.54 ± 0.05	2.79 ± 0.03	740.35 ± 0.02
15	32.65 ± 0.23	4.50 ± 0.12	873.55 ± 0.01
30	77.38 ± 0.19	10.09 ± 0.10	2059.02 ± 0.04
Pore II			
3.75	11.90 ± 0.04	1.78 ± 0.02	321.11 ± 0.05
7.5	20.43 ± 0.06	2.92 ± 0.04	548.64 ± 0.01
15	32.36 ± 0.09	4.50 ± 0.05	866.69 ± 0.01
30	65.38 ± 0.45	10.74 ± 0.02	1783.93 ± 0.01

Based on the cell tolerance test for L929 and BEC, bFGF content of 3.75 ng with Pore II scaffolds was chosen for subsequent testing, as the average single-point release concentration never exceeds 0.05 ng/mL, while the cumulative release total after 21 days is 321.11 pg. These conditions result in slow release, yet the concentrations do not rise into overdose regimes.

### 3.5. Controlled release of transforming growth factor β1

**Figure 7.**
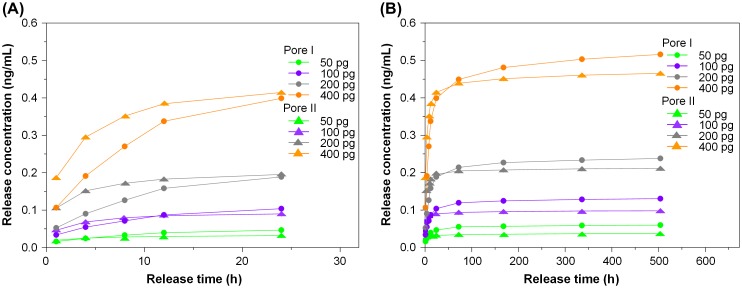
The cumulative release of various TGFβ1 contents from porous chitosan scaffolds within (a) 24 h and (b) up to 21 days (1, 4, 8, 12, 24 h, and 3, 7, 14, 21 days)

**Figure 8.**
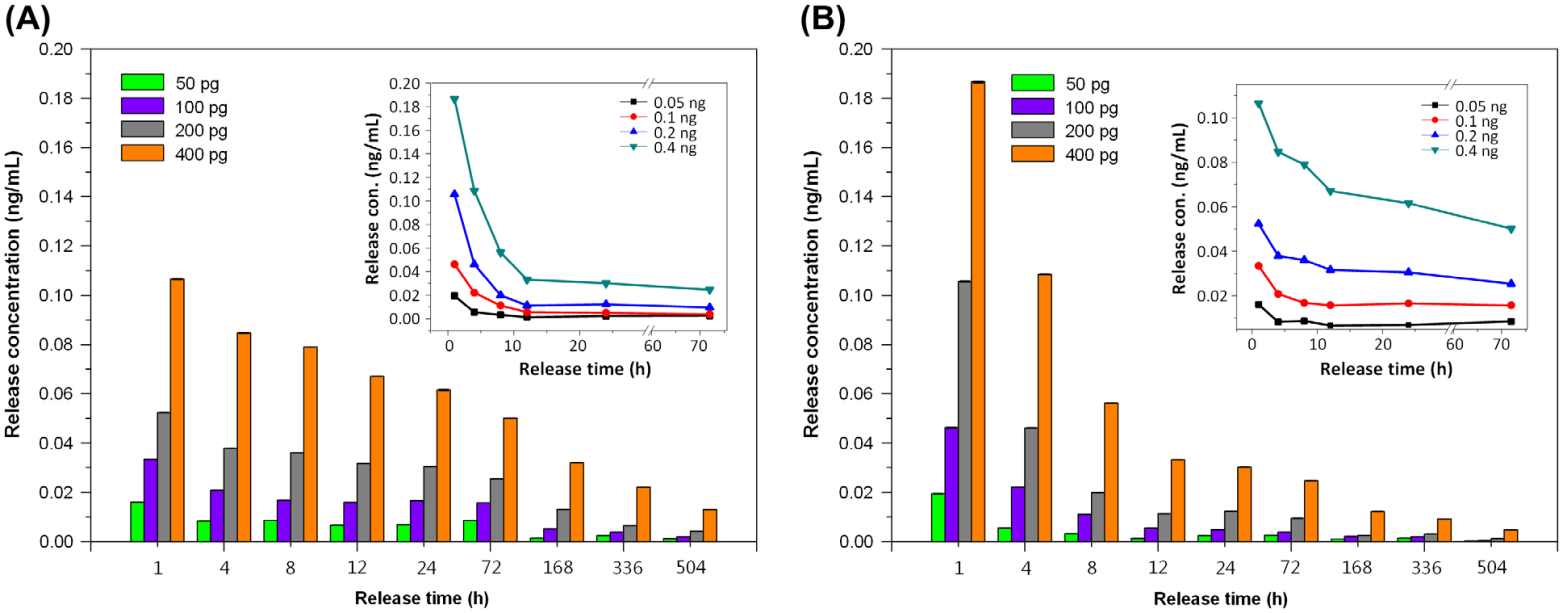
Single-point release of different TGFβ1 content at 1, 4, 8, 12, 24, 72, 168, 336, and 504 h for (a) Pore I and (b) Pore II chitosan scaffolds. Inset: reduction trends for different loadings.

The general trend observed in the release profile of TGFβ1 is similar to that seen in bFGF. The cumulative release profile and single-point release of different TGFβ1 contents are shown in Figures 7 and 8, respectively, for chitosan scaffolds, Pore I and Pore II. The reduction trends for different loadings are shown in the inset of Figure 8. It was found that TGFβ1 was released in large quantities within the first 24 h and reached steady state on the third day, regardless of the initial content and type of scaffold. It was observed that the amount released by Pore I scaffolds was greater than Pore II, and Table 2 summarizes the release characteristics of both scaffolds. With an initial TGFβ1 content of 400 pg, the average release within the first 24 h was 8.31 and 8.64 pg/h for Pore I and Pore II scaffolds, respectively, which accounts for 2.08% and 2.16% of the total TGFβ1 content. This result may have been due to the surface TGFβ1 releasing rapidly within the first few hours. As the release time progressed, it could be seen that the concentration of TGFβ1 within the culture medium decreased and reached steady state by the third day, with an average TGFβ1 release rate of 2.93 pg/day and 1.27 pg/day for chitosan scaffolds Pore I and Pore II, respectively. The cumulative total of TGFβ1 released was calculated after 21 days (504 h), and it was found that Pore I released a total of 258.02 pg (64.51% of total), while Pore II scaffolds released a total of 232.63 pg (58.16% of total). The results are consistent with previous findings that large pore sizes will enable high diffusion rates. In addition, the release rate of TGFβ1 was observed to be significantly less than that of bFGF; this is due to the initial amount of growth factor content within the scaffolds; however, it was found that after 21 days, scaffolds released more than half of the initial TGFβ1 content. In Tables 1 and 2, it is observed that an increase in the initial growth factor content will increase the amount released, due to an increased initial diffusion rate. This indicates that the controlling release mechanism is thus likely diffusion.

**Table 2.** Release characteristics of various TGFβ1 contents from porous chitosan scaffolds.

bFGF content (ng)	Average release within 24 h (pg/h)	Average release from day 1 to day 21 (pg/day)	Cumulative total over 21 days (pg)
Pore I			
50	0.97 ± 0.02	0.33 ± 0.01	29.88 ± 0.01
100	2.16 ± 0.06	0.67 ± 0.04	65.12 ± 0.09
200	3.93 ± 0.02	1.23 ± 0.02	118.88 ± 0.04
400	8.31 ± 0.02	2.93 ± 0.01	258.02 ± 0.03
Pore II			
50	0.66 ± 0.03	0.13 ± 0.02	18.40 ± 0.09
100	1.87 ± 0.01	0.21 ± 0.01	48.98 ± 0.01
200	4.06 ± 0.07	0.40 ± 0.04	105.64 ± 0.02
400	8.64 ± 0.01	1.27 ± 0.06	232.63 ± 0.02

From the results summarized in Table 2, the average release rate of Pore I scaffolds containing 50 and 100 pg of TGFβ1 after 21 days did not exceed the concentration limit of 1 pg/mL. Similarly, the release rate of Pore II scaffolds containing 50, 100, and 200 pg after 12 h did not exceed the concentration limit of 1 pg/mL, which was determined in the cell tolerance tests. Pore I scaffolds with 400 pg of TGFβ1 were chosen for subsequent cell experimentation to evaluate biodegradable porous chitosan scaffolds as effective drug release carriers with inherent dosage control.

### 3.6. Effect of porous chitosan scaffolds containing bFGF and TGFβ1 on L929 and BEC proliferation

In this study, porous chitosan scaffolds were used to investigate the effect of released growth factors on the proliferation of fibroblasts and endothelial cells. The experiment groups used were 3.75 ng bFGF containing Pore II scaffolds, 400 pg TGFβ1 containing Pore I scaffolds, and also non-growth factor containing Pore I and II scaffolds, to evaluate the cell proliferation after seven days of L929 and BEC culture; the cell viability is displayed as a percentage relative to control culture on TCPS.

**Figure 9.**
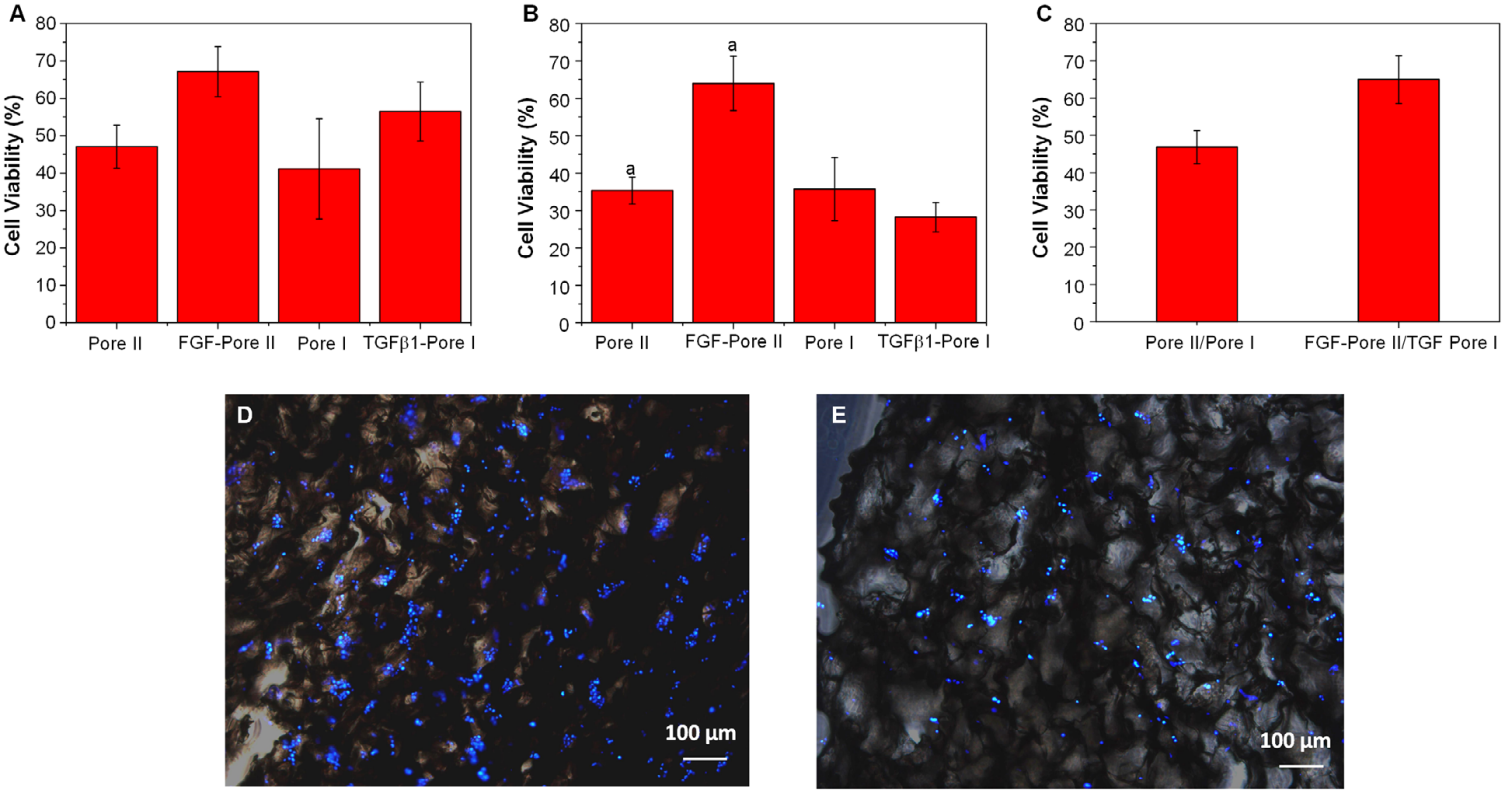
Effect of porous chitosan scaffolds on the proliferation of (a) L929 and (b) BEC in the presence and absence of growth factors (bFGF and TGFβ1) relative to TCPS. Letter *a* in (a) indicates a significant difference; (c) Cell viability of L929 and BEC co-culture in Pore I and Pore II scaffolds containing TGFβ1 and bFGF, respectively, relative to TCPS. (*p* < 0.05). Fluorescent images of (d) L929 and (e) BEC cultured on porous chitosan scaffolds containing growth factors bFGF and TGFβ1. Blue represents nucleus on the chitosan scaffold.

The choice of porous chitosan scaffolds as wound dressings has advantages such as good porosity, suitable growth substrate for cells, and are able to absorb large amounts of wound exudates [21]. The results of continuous culture of L929 cells for seven days using chitosan scaffolds containing bFGF and TGFβ1 are shown in Figure 9(a). It was found that the cell proliferation on scaffolds containing bFGF (Pore II) and TGFβ1 (Pore I) was 1.43 and 1.37 times greater than their respective pure scaffolds (no growth factor loaded). This result indicates that both bFGF and TGFβ1 can enhance the proliferation of L929, with bFGF having a greater effect, as this growth factor has been known to stimulate fibroblast proliferation and differentiation, whereas the main function of TGFβ1 is to stimulate collagen production. Similarly, Figure 9(b) illustrates the proliferation of BEC cultured within porous chitosan scaffolds with and without growth factors present. In Pore II scaffolds with bFGF, it was found that cell viability was 1.81-fold greater than pure porous scaffolds, while cell proliferation in TGFβ1 (Pore I) was 0.79 times less than pure scaffold. The results observed are consistent with previous results from cell tolerance analysis of bFGF and TGFβ1 effects on L929 and BEC proliferation.

The co-culture of L929 and BEC with bFGF-Pore II and TGFβ1-Pore I scaffolds was conducted to investigate the potential for dual drug-releasing scaffolds (Figure 9(c)). It was found that the cells co-cultured on scaffolds containing growth factors had 1.39 times greater proliferation and viability than the scaffolds without added growth factors. The cell distribution on growth factor contained scaffold was shown in Figure 9(d) and (e). Cells were uniformly distributed on the scaffold, and some cells were submerged below the scaffold. This implies that the porous scaffold contains connected pore, which is beneficial for cell migration, proliferation, and metabolism. This result suggests that there is potential for further investigation into porous chitosan scaffolds that enable multiple drug release that affects multiple cell type cultures with applications in wound healing.

In all of the experiments, it was found that the proliferation rates and viability of cells cultured on chitosan scaffolds were lower than that on TCPS culture (i.e. cell viability is less than 100%), this observation can be attributed to commercial TCPS with coatings that allow for greater attachment and proliferation, whereas chitosan is a hydrophobic material, which results in lower cell affinity. It has been reported that hydrogen bonding, electrostatic force, or polarity of the culture substrate will affect cell activity and viability [32].

## 4. Conclusions

In this study, chitosan scaffolds with the ability to control the drug release rate were successfully fabricated via a freeze-drying method with varying temperature and times, which can control the pore sizes of the scaffolds. This study investigated the drug release of scaffolds with pores sizes of 34 ± 9 μm (Pore II) and 153 ± 32 μm (Pore I). It was found that after 21 days, the total amount of drug released by Pore I scaffolds was greater than that of Pore II due to large pore sizes facilitating greater diffusion rates. In addition, the results show that the drug release could be sustained over a long period of time due to the use of porous scaffolds. The results also demonstrated that 3.75 ng of bFGF in Pore II scaffolds is able to promote the proliferation of L929 fibroblast and BEC cells. It was also found that 400 pg of TGFβ1 in Pore I scaffolds can promote L929 proliferation, but could also inhibit BEC expansion. This study paves the way for the development of a dual drug-releasing biocompatible chitosan scaffold, such as bFGF and TGFβ1, to effectively control the wound healing process via the regulation of specific cell proliferation.

## Disclosure statement

No potential conflict of interest was reported by the authors.

## Funding

This work was supported by Ministry of Science, Technology of Taiwan [grant number MOST-104-2923-E-005-001-MY2]; the Ministry of Science, Technology and Space, Israel [grant number 3-12407].
